# Study on the Magnetic-machine Coupling Characteristics of Giant Magnetostrictive Actuator Based on the Free Energy Hysteresis Characteristics

**DOI:** 10.3390/s18093070

**Published:** 2018-09-12

**Authors:** Zhen Yu, Tao Wang, Min Zhou

**Affiliations:** 1 Key Laboratory of Metallurgical Equipment and Control Technology of Ministry of Education, Wuhan University of Science and Technology, Wuhan 430081, China; 2 Hubei Key Laboratory of Mechanical Transmission and Manufacturing Engineering, Wuhan University of Science and Technology, Wuhan 430081, China; wangtao77@wust.edu.cn; 3 Hubei Wuhan Tobacco Company Cigarette Logistics Distribution Center, Wuhan 430000, China; zm.323@163.com

**Keywords:** giant magnetostrictive actuator, free energy, hysteresis characteristics, coupling characteristics of magnetic and machine, COMSOL simulation

## Abstract

A giant magnetostrictive actuator presents advantages such as large strain, high precision, and quick response. It is a hotly debated research topic in the field of micro drivers; however, the nonlinear intrinsic relationship between its output and input signals make it difficult to construct its nonlinear eigen model in the process of its practical application. Therefore, the motivation of this paper is to study the nonlinear magnetic–mechanical coupling characteristics of the giant magnetostrictive actuator, which is driven by free energy hysteresis characteristics. The nonlinear magnetic–mechanical coupling model under the weak form solution is deduced from the basic electromagnetic and mechanical theories, based on the distribution law of the axial magnetic field simulation, carried out to analyze the output displacement characteristics of the giant magnetostrictive actuator under preload. Experimental characterization of the device is also studied in the built experiment setup. Research results show that the experimental results coincide well with the simulation results, which show that the designed magnetic circuit for the giant magnetostrictive actuator is correct, and the coupling model of magnetic and machine of the giant magnetostrictive actuator based on the free energy hysteresis characteristics is reasonable.

## 1. Introduction

At present, the functional materials used in the field of micro-actuation technology mainly include piezoelectric materials, shape memory alloys, and giant magnetostrictive materials [1,2,3,4,5,6].

Based on the giant magnetostrictive (GMS) effect, a novel giant magnetostrictive actuator with high actuation power is designed and implemented by Mingzhang Luo [7]. This design enables the generation of stress waves with high energy, and the focusing of the generated stress waves on the test object, it can be used in the quality assessment of rock bolt-reinforced structures and other nondestructive testing and evaluation applications that require high-power stress wave generation. Zhenyuan Jia et al. [8] used a giant magnetostrictive actuator (GMA) made of giant magnetostrictive material (GMM) to apply a magnetic field to strain it by using its magnetostrictive effect and to produce output force or displacement using its longitudinal deformation, with large strain, high precision, fast response, high reliability, and so on. It quickly became a research hotspot in the field of micro-drive. Yuanyuan Yang and his college [9] present an induced voltage linear extraction method for disturbing force self-sensing in the application of giant magnetostrictive actuators, a Kelvin bridge combined with an active device is constructed instead of a conventional Wheatstone bridge for extraction of the induced voltage, the method for solving the nonlinear problems in GMA self-sensing signal extraction has been demonstrated. Mingzhang Luo [10] attempts to develop a portable, non-destructive evaluation method for assessing the length of installed rock bolts for inspection purposes, we proposed a portable device for the non-destructive evaluation of rock bolt conditions based on a giant magnetostrictive (GMS) actuator. The GMS actuator generates enough energy to ensure multiple reflections of the stress waves along the rock bolt and a lead zirconate titantate (PZT) sensor is used to detect the reflected waves. This paper can also be adopted for property assessment of others to accurately determine the rock bolt length. At present, GMA has reported on the application of precision fluid transmission and control, precision machining, precision peristaltic mechanism, sonar, vibration suppression, measurement and control, and so on.

Research [8,11,12,13] shows the following points: There is a nonlinear eigen relation between the output signal and the input signal of the GMA. It is a major problem in the practical application of intelligent materials for its intrinsic nonlinearity. Because of the strong coupling property between the material itself and the electromagnetic heat, it is very complicated for the nonlinear problem, which in turn makes modeling very difficult. In the research of a multi-physics dynamic magnetic-mechanical coupling model, scholars at home and abroad have also done many analyses and much research.

In the research by Yongxin Guo [14], a new Hammerstein model is proposed for modeling the rate-dependent hysteresis nonlinearity of a GMA, the proposed Hammerstein model can describe the hysteresis loops well within a frequency range of 0–100 Hz. However, based on this model, only a 2 DOF (DOF is an abbreviation for degree of freedom) control system is designed.

A novel adaptive filter is proposed to model the rate-dependent hysteresis nonlinearity in a giant magnetostrictive actuator [15]. In the proposed filter, generalized play operators are combined with linear delayed adaptive transversal filter to compose a new serial structure of adaptive filter model. The proposed adaptive filter is applied to model the rate dependent hysteresis of giant magnetostrictive actuator. The experimental results show that the proposed generalized play operator adaptive filter can describe the rate-dependent hysteresis behaviors including different single frequency input signal and multi-frequency composite input signal.

A.E. Clark [16] has proposed a linear piezomagnetic constitutive equation for a giant magnetostrictive actuator based on a large number of studies, this equation accurately describes the magnetic–mechanical coupling relationship of giant magnetostrictive materials, making it a magnetic–machine coupling basic equations in the study of giant magnetostrictive actuators.

As early as 1995, M.E.H. Benbouzid et al. [17,18] used the finite element simulation model to analyze the nonlinear dynamic characteristics of the giant magnetostrictive rod, which can guide the optimization of the magnetic circuit design of the giant magnetostrictive actuator. However, the model is a two-dimensional planar model, and its dynamic characteristics are obtained from the static characteristics of giant magnetostrictive materials. Because static modeling has many parameters, the calculation is complex and susceptible to other field-related parameters; therefore, the model itself has certain limitations.

Azoum [19] established a three-dimensional generalized finite element model based on the coupled constitutive equation of the giant magnetostrictive actuator, and simulated the solenoid coil. Simultaneously, Benatar [20] established a three-dimensional electro-magnetic-mechanical coupling model of GMM transducers used the multiphysics coupling software FEMLAB (the predecessor of COMSOL software which eveloped by the COMSOL group who founded in Stockholm, Sweden). Zhao Zhangrong and others from Zhejiang University [21] also used the weak solution of COMSOL software to simulate and analyze the electro–magnetic–mechanical coupling model of intelligent mast components of GMM, but the coefficient matrix substituted is a constant coefficient matrix, Therefore, the simulation result obtained is linear, and there is a certain error with the experimental results.

None of the above methods establishes a model based on the characteristics of the giant magnetostrictive material itself, and the giant magnetostrictive material is a ferromagnetic material, its essential feature is that there are intrinsic nonlinearities and hysteresis characteristics, so it is a reasonable method to study the intrinsic relationship between the output and input of the actuator based on the intrinsic nonlinearity and hysteresis characteristics of the giant magnetostrictive material.

In this paper, the hysteresis characteristics and nonlinearity of giant magnetostrictive actuators are studied in depth by the free energy hysteresis model. The three-dimensional magnetic machine coupling model of the giant magnetostrictive actuator is established, and a three-dimensional nonlinear coupled model of the giant magnetostrictive actuator is established according to the free-energy hysteresis model that used the material eigen model combined with the three-dimensional coupled model, then the finite element simulation software is used for analysis and calculation to obtain the magnetic induction and strain increments. Following this, the total strain output is obtained, and the simulation results were verified by experiments.

## 2. The Theoretical Basis of the Magnetic Machine Coupling Model for the Giant Magnetostrictive Actuator (GMA)

### 2.1. The Fundamental Theory of the Magnetic Field Excited by the Winding for GMA

For the giant magnetostrictive actuator system, Maxwell’s equations [22,23,24] are used to solve the distribution of the magnetic field excited by the winding. The solving variables are set as the vector magnetic potential ($A_{x},A_{y},A_{z}$) in the magnetic field. According to the differential Maxwell equations of the magnetic field and the principle of current continuity, the driving frequency of the magnetic field excited by the winding is lower than 30 MHz. The driving current source is chosen to be a constant current source so that the magnetic field is formed to be a constant magnetic field. Therefore, the electric displacement field vector D can be neglected, then $\frac{\partial D}{\partial t}=0$. The total current density consists of two parts: source current density $J_{S}$ and applied current density $J_{E}$, which is caused by eddy current effect, then the divergence of magnetic field intensity is as follows:

(1) $$\nabla \times H=J_{S}+J_{E}$$

In this equation, H denotes the magnetic field intensity vector.

Electric field intensity E can be expressed by magnetic vector potential:

(2) $$E=-\frac{\partial A}{\partial t}-\nabla \phi$$

where ϕ is a scalar potential function.

Then the applied current density caused by eddy current effect is expressed as (3):

(3) $$J_{E}=\sigma E=\sigma (-\frac{\partial A}{\partial t}-\nabla \phi )=\sigma (-\frac{\partial A}{\partial t}-0)=-\sigma \frac{\partial A}{\partial t}$$

where σ is electric conductivity.

Then the magnetic flux density B is expressed by magnetic vector potential:

(4) $$B=\nabla \times A=\left|\begin{array}{ccc} i & j & k\\ \frac{\partial }{\partial x} & \frac{\partial }{\partial y} & \frac{\partial }{\partial z}\\ A_{x} & A_{y} & A_{z} \end{array}\right|=(\frac{\partial A_{z}}{\partial y}-\frac{\partial A_{y}}{\partial z})i+(\frac{\partial A_{x}}{\partial z}-\frac{\partial A_{z}}{\partial x})j+(\frac{\partial A_{y}}{\partial x}-\frac{\partial A_{x}}{\partial y})k$$

### 2.2. The Fundamental Theory of the Mechanical Field of GMA

According to Newton’s Second Law, the motion of a giant magnetostrictive rod can be described as follows:

(5) $$\nabla \cdot T+f_{B}=m\frac{d^{2}u}{dt^{2}}$$

where the displacement $u={\left[\begin{array}{ccc} u_{x} & u_{y} & u_{z} \end{array}\right]}^{T}$, the stress $T=\left[\begin{array}{ccc} T_{1} & T_{12} & T_{13}\\ T_{21} & T_{2} & T_{23}\\ T_{31} & T_{32} & T_{3} \end{array}\right]$, $T_{12}=T_{21}$, $T_{13}=T_{31}$, $T_{23}=T_{32}$, and $f_{B}$ is body force.

When damping is considered, and Navier-stocks equation [23] is combined to derive Formula (6):

(6) $$\nabla \cdot T+f_{B}=m\frac{d^{2}u}{dt^{2}}+c\frac{\partial u}{\partial t}$$

where c is the viscous damping coefficient, and m is quality of the system.

The solving variable of the mechanical field is set as displacement vector ($u_{x},u_{y},u_{z}$), then the relationship between the elastic strain S and the displacement can be expressed as follows:

(7) $$S=\nabla^{s}u$$

where $\nabla^{s}={\left[\begin{array}{cc} \begin{array}{ccc} \frac{\partial }{\partial x} & 0 & 0\\ 0 & \frac{\partial }{\partial y} & 0\\ 0 & 0 & \frac{\partial }{\partial z} \end{array} & \begin{array}{ccc} \frac{\partial }{\partial y} & 0 & \frac{\partial }{\partial z}\\ \frac{\partial }{\partial x} & \frac{\partial }{\partial z} & 0\\ 0 & \frac{\partial }{\partial y} & \frac{\partial }{\partial x} \end{array} \end{array}\right]}^{T}$. If it is substituted into Equation (7), Formula (8) is obtained as follows:

(8) $$\left[\begin{array}{c} S_{1}\\ S_{2}\\ S_{3}\\ S_{12}\\ S_{23}\\ S_{13} \end{array}\right]=\nabla^{s}u={\left[\begin{array}{cc} \begin{array}{ccc} \frac{\partial }{\partial x} & 0 & 0\\ 0 & \frac{\partial }{\partial y} & 0\\ 0 & 0 & \frac{\partial }{\partial z} \end{array} & \begin{array}{ccc} \frac{\partial }{\partial y} & 0 & \frac{\partial }{\partial z}\\ \frac{\partial }{\partial x} & \frac{\partial }{\partial z} & 0\\ 0 & \frac{\partial }{\partial y} & \frac{\partial }{\partial x} \end{array} \end{array}\right]}^{T}\left[\begin{array}{c} u_{x}\\ u_{y}\\ u_{z} \end{array}\right]$$

### 2.3. The Conversion of the Weak Form Solution

(1)   Einstein notation

According to Einstein Notation [24], tensor $\in_{ijk}$ is a Levi–Civita signal, which is defined as follows:

(9) $$\in_{ijk}=\{\begin{array}{l} +1,\;\;\;if(i,j,k)=(1,2,3),(2,3,1)or(3,1,2);\\ -1,\;\;\;if(i,j,k)=(3,2,1),(2,1,3)or(1,3,2);\\ 0,\;\;\;\;\;\;i=j,j=k,or,k=i; \end{array}$$

The cross product of the two variables can be expressed as follows:

(10) $${\left(a\times b\right)}_{i}=\in_{ijk}a_{j}b_{k}$$

The curl of the variables is shown as follows:

(11) $${\left(\nabla \times a\right)}_{i}=\in_{ijk}\frac{\partial a_{k}}{\partial x_{j}}$$

Use Einstein Notation to mark Equations (1) and (3):

(12) $$\begin{array}{l} {\left(\nabla \times H\right)}_{i}={\left(J_{S}\right)}_{i}+{\left(J_{E}\right)}_{i}\\ {\left(J_{E}\right)}_{i}=(-\sigma \frac{\partial A}{\partial t}) \end{array}$$

Substituting Equation (11) into the above two equations:

(13) $$\in_{ijk}\frac{\partial H_{k}}{\partial x_{j}}={\left(J_{S}\right)}_{i}+\left(-\sigma \frac{\partial A}{\partial t}\right){}_{i}={\left(J_{S}\right)}_{i}-\sigma \frac{\partial A_{i}}{\partial t}$$

Use Einstein Notation to mark Equation (6) and expand the formula:

(14) $${\left(\nabla \cdot T\right)}_{i}+{\left(f_{B}\right)}_{i}=\left(m\frac{d^{2}u}{dt^{2}}\right){}_{i}+\left(c\frac{\partial u}{\partial t}\right){}_{i}$$

(15) $$\frac{\partial T_{ij}}{\partial x_{j}}+{\left(f_{B}\right)}_{i}=m\frac{d^{2}u_{i}}{dt^{2}}+c\frac{\partial u_{i}}{\partial t}$$

Use weighted residual method to integrate Equations (13) and (15):

(16) $$\begin{array}{l} \int_{V_{B}}\in_{ijk}\frac{\partial H_{k}}{\partial x_{j}}\psi_{i}dV+\int_{V_{B}}\sigma \frac{\partial A_{i}}{\partial t}\psi_{i}dV=\int_{V_{B}}{\left(J_{S}\right)}_{i}\psi_{i}dV\\ \int_{V_{u}}\frac{\partial T_{ij}}{\partial x_{j}}\phi_{i}dV+\int_{V_{u}}{\left(f_{B}\right)}_{i}\phi_{i}dV=\int_{V_{u}}m\frac{d^{2}u_{i}}{dt^{2}}\phi_{i}dV+\int_{V_{u}}c\frac{\partial u_{i}}{\partial t}\phi_{i}dV \end{array}$$

where $\psi_{i}$ and $\phi_{i}$ are weight function, $V_{B}$ is magnetic field distribution region, and $V_{u}$ is action area of mechanical field.

Take partial integration for the first term on the left of the Equation (16), then Formula (17) is derived as:

(17) $$\begin{array}{l} \int_{V_{B}}\in_{ijk}\frac{\partial H_{k}}{\partial x_{j}}\psi_{i}dV=\int_{V_{B}}\in_{ijk}\frac{\partial (H_{k}\psi_{i})}{\partial x_{j}}dV-\int_{V_{B}}\in_{ijk}H_{k}\frac{\partial \psi_{i}}{\partial x_{j}}dV\\ \int_{V_{u}}\frac{\partial T_{ij}}{\partial x_{j}}\phi_{i}dV=\int_{V_{u}}\frac{\partial (T_{ij}\phi_{i})}{\partial x_{j}}dV-\int_{V_{u}}T_{ij}\frac{\partial \phi_{i}}{\partial x_{j}}dV \end{array}$$

Define the divergence $\int_{V}^{}\nabla \cdot FdV=\int_{S}^{}F\cdot ndS$, according to divergence theorem, the first term on the right of the Equation (17) can be expressed as follows:

(18) $$\begin{array}{l} \int_{V_{B}}\in_{ijk}\frac{\partial (H_{k}\psi_{i})}{\partial x_{j}}dV=\int_{\partial V_{B}}\in_{ijk}H_{k}\psi_{i}n_{j}d\partial V\\ \int_{V_{u}}\frac{\partial (T_{ij}\phi_{i})}{\partial x_{j}}dV=\int_{\partial V_{u}}T_{ij}\phi_{i}n_{j}d\partial V \end{array}$$

Substitute the above Equation into equation (17):

(19) $$\begin{array}{l} \int_{V_{B}}\in_{ijk}\frac{\partial H_{k}}{\partial x_{j}}\psi_{i}dV=\int_{\partial V_{B}}\in_{ijk}H_{k}\psi_{i}n_{j}d\partial V-\int_{V_{B}}\in_{ijk}H_{k}\frac{\partial \psi_{i}}{\partial x_{j}}dV\\ \int_{V_{u}}\frac{\partial T_{ij}}{\partial x_{j}}\phi_{i}dV=\int_{\partial V_{u}}T_{ij}\phi_{i}n_{j}d\partial V-\int_{V_{u}}T_{ij}\frac{\partial \phi_{i}}{\partial x_{j}}dV \end{array}$$

Then the Equation (19) can be rearranged as follows:

(20) $$\begin{array}{l} \int_{V_{B}}\in_{ijk}\frac{\partial H_{k}}{\partial x_{j}}\psi_{i}dV=-\int_{\partial V_{B}}\in_{ijk}\in_{ijk}H_{j}n_{k}\psi_{i}d\partial V+\int_{V_{B}}\in_{ijk}H_{i}\frac{\partial \psi_{k}}{\partial x_{j}}dV\\ \int_{V_{u}}\frac{\partial T_{ij}}{\partial x_{j}}\phi_{i}dV=\int_{\partial V_{u}}T_{ij}\phi_{i}n_{j}d\partial V-\int_{V_{u}}T_{ij}\frac{\partial \phi_{i}}{\partial x_{j}}dV \end{array}$$

Substitute the above equation into Equation (16):

(21) $$\begin{array}{l} \int_{V_{B}}\in_{ijk}H_{i}\frac{\partial \psi_{k}}{\partial x_{j}}dV+\int_{V_{B}}\sigma \frac{\partial A_{i}}{\partial t}\psi_{i}dV=\int_{\partial V_{B}}\in_{ijk}H_{j}n_{k}\psi_{i}d\partial V+\int_{V_{B}}{\left(J_{S}\right)}_{i}\psi_{i}dV\\ \int_{V_{u}}m\frac{d^{2}u_{i}}{dt^{2}}\phi_{i}dV+\int_{V_{u}}c\frac{\partial u_{i}}{\partial t}\phi_{i}dV+\int_{V_{u}}T_{ij}\frac{\partial \phi_{i}}{\partial x_{j}}dV=\int_{\partial V_{u}}T_{ij}\phi_{i}n_{j}d\partial V+\int_{V_{u}}{\left(f_{B}\right)}_{i}\phi_{i}dV \end{array}$$

According to Galerkin Method [22,24], weight function is equal to basis function, where minimal variables $A_{i},\;u_{i}$ are used. The weight function is expanded to the following approximate expression:

(22) $$\begin{array}{l} \psi_{i}=\delta A_{i}\\ \phi_{i}=\delta u \end{array}$$

Equation (21) can be expressed as follows:

(23) $$\begin{array}{l} \int_{V_{B}}\in_{ijk}H_{i}\frac{\partial \delta A_{k}}{\partial x_{j}}dV+\int_{V_{B}}\sigma \frac{\partial A_{i}}{\partial t}\delta A_{i}dV=\int_{\partial V_{B}}\in_{ijk}H_{j}n_{k}\delta A_{i}d\partial V+\int_{V_{B}}{\left(J_{S}\right)}_{i}\delta A_{i}dV\\ \int_{V_{u}}m\frac{d^{2}u_{i}}{dt^{2}}\delta u_{i}dV+\int_{V_{u}}c\frac{\partial u_{i}}{\partial t}\delta u_{i}dV+\int_{V_{u}}T_{ij}\frac{\partial \delta u_{i}}{\partial x_{j}}dV=\int_{\partial V_{u}}T_{ij}\delta u_{i}n_{j}d\partial V+\int_{V_{u}}{\left(f_{B}\right)}_{i}\delta u_{i}dV \end{array}$$

This is the weak solution equation for solving the magnetic and mechanical field variables of GMA system under the Einstein Notation.

(2)   Matrix notation

Equation (23) can be expressed by matrix notation as Equation (24):

(24) $$\begin{array}{l} \int_{V_{B}}H\cdot (\nabla \times \delta A)dV+\int_{V_{B}}\sigma \frac{\partial A}{\partial t}\delta AdV=\int_{V_{B}}J_{S}\delta AdV+\int_{\partial V_{B}}(H\times n)\cdot \delta Ad\partial V\\ \int_{V_{u}}m\frac{d^{2}u}{dt^{2}}\delta udV+\int_{V_{u}}c\frac{\partial u}{\partial t}\delta udV+\int_{V_{u}}T\cdot \nabla \delta udV=\int_{\partial V_{u}}(T\cdot n)\delta ud\partial V+\int_{V_{u}}f_{B}\delta udV \end{array}$$

The equations for the weak solution formally express the balance of virtual work both inside and outside the system. According to the kinematics relationship, the variation variables δB=∇×δA, δS=∇δu are introduced to calculate H and T. Because the surface traction at the mechanical field boundaries is t=Tn, the tangential component of the magnetic field boundaries is $H_{T}=H\times n$, then the virtual work of the system can be expressed as follows:

(25) $$\begin{array}{l} \int_{V_{B}}H\cdot \delta BdV+\int_{V_{B}}\sigma \frac{\partial A}{\partial t}\delta AdV=\int_{V_{B}}J_{S}\delta AdV+\int_{\partial V_{B}}H_{T}\cdot \delta Ad\partial V\\ \int_{V_{u}}m\frac{d^{2}u}{dt^{2}}\delta udV+\int_{V_{u}}c\frac{\partial u}{\partial t}\delta udV+\int_{V_{u}}T\cdot \delta SdV=\int_{\partial V_{u}}t\cdot \delta ud\partial V+\int_{V_{u}}f_{B}\delta udV \end{array}$$

### 2.4. Three-Dimensional Finite Element Discrimination of Virtual Work Model for the System

Using the finite element method, the solvability domain is discretized into a finite number of units (the number of element is assumed to be e), the weak form solution Equation (24) is solved on the element. The solving variables, the vector potential energy, offset variables of every element should be calculated by node value interpolation. Thus, their interpolation will be written into matrix form using the interpolation and the shape functions to calculate the variable node value.

Interpolation and integration are calculated at the local coordinate ξ, which is combined with dx and dξ through the Jacobian matrix ${𝒥}_{e}$, where $dx={𝒥}_{e}d\xi$, $dV=\det({𝒥}_{e})d\xi_{1}d\xi_{2}d\xi_{3}=J_{e}d\xi_{1}d\xi_{2}d\xi_{3}$. For the linear interpolation of geometry structure, the variables $A_{e}$ and $u_{e}$ of each element can be interpolated by node values $q_{e}^{A}$ and $q_{e}^{u}$:

(26) $$\begin{array}{l} A_{e}=N_{A}(\xi )q_{e}^{A}\\ u_{e}=N_{u}(\xi )q_{e}^{u} \end{array}$$

According to the Galerkin method [23,24], weight function is equal to basis function, then the shape function of minimal variables is written as follows:

(27) $$\begin{array}{l} \delta A_{e}=N^{A}(\xi )\delta q_{e}^{A}\\ \delta u_{e}=N^{u}(\xi )\delta q_{e}^{u} \end{array}$$

Because A is a three-dimensional vector, the interpolating matrix $N^{A}$ presents three lines, $N_{n}^{A}$ nods in each element ($N_{n}^{A}$ is depended on the order of the element), and every nod can be described as a three-dimensional vector, which is relative with the nodal value $A_{e}$. So the interpolating matrix $N^{A}$ consists of $N_{{}^{q}}^{A}$ columns, where $N_{{}^{q}}^{A}=3N_{n}^{A}$. The dimension of the vector $q_{e}^{A}$ is $N_{{}^{q}}^{A}$, and all three components in each node are $A_{e}$. Because the displacement variables does not require the same shape function, the available number of columns of $N^{u}$ can be expressed by $N_{q}^{u}$, which depended on the number of node $N_{n}^{u}$. From the above information, it is observed that the total degree of freedom of each element is $N_{q}=N_{q}^{u}+N_{q}^{A}$.

**Figure 1:**
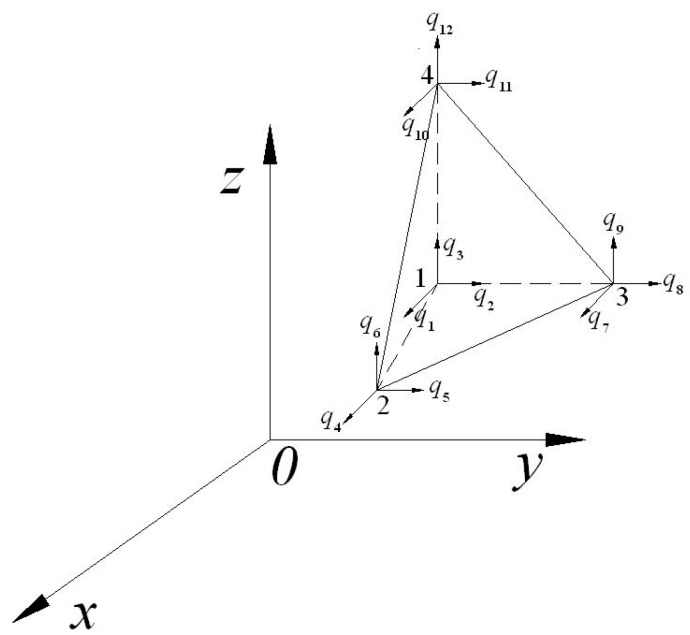
Four node tetrahedral element and its coordinate system.

In this paper, the typical shape functions are mainly tetrahedron elements of linear functions or quadratic Lagrange function [24]. Assuming that vector potential energy and displacement variables are expressed in four-node tetrahedral element (as shown in Figure 1), then $N^{A}=N^{q}=N$. Matrix shape function N is consisted of a Lagrange function:

(28) $$\begin{array}{l} N_{1}=\xi_{1}\\ N_{2}=\xi_{2}\\ N_{3}=\xi_{3}\\ N_{4}=1-\xi_{1}-\xi_{2}-\xi_{3}, \end{array}$$

where, for node i, when j=i, $N_{j}=1$, otherwise, when j≠i, $N_{j}=0$. According to the shape function (28), in the global coordinate system, $x_{1}$ can be interpolated with the node value $x_{1,n}$.

(29) $$x_{1}=N_{1}x_{1,1}+N_{2}x_{1,2}+N_{3}x_{1,3}+N_{4}x_{1,4}$$

Because the four-node tetrahedral element in the finite elements is used, the dimension of vector $q_{e}^{A}$ is 12, and the first three dimensions at node 1 are the three components of $A_{e}$, which is followed by the three components at nodes 2, 3, and 4. The matrix of shape function is shown as Formula (30).

(30) $$N(\xi )=\left[\begin{array}{cccc} \begin{array}{ccc} N_{1} & 0 & 0\\ 0 & N_{1} & 0\\ 0 & 0 & N_{1} \end{array} & \begin{array}{ccc} N_{2} & 0 & 0\\ 0 & N_{2} & 0\\ 0 & 0 & N_{2} \end{array} & \begin{array}{ccc} N_{3} & 0 & 0\\ 0 & N_{3} & 0\\ 0 & 0 & N_{3} \end{array} & \begin{array}{ccc} N_{4} & 0 & 0\\ 0 & N_{4} & 0\\ 0 & 0 & N_{4} \end{array} \end{array}\right]$$

When local coordinate ξ=(1,0,0) is located at node 1, $A_{e}=A_{e,1}$, the vector potential energy can be simplified to the value of node 1. Similarly, when local coordinate ξ=(0,1,0) is located at node 2, $A_{e}=A_{e,2}$, the vector potential energy can be simplified to the value of node 2. It is the same for the other coordinates. For other points in the tetrahedron, generally, the linear interpolation of node values is used for the shape function; for instance, local coordinates is written as ξ=(1/2,1/2,0), $A_{e}=1/2(A_{e,1}+A_{e,2})$.

In the finite element model, the node values of the vector potential energy and the displacement variables are unknown. The virtual vector potential energy and the displacement variables can be set to be any values. In order to show finite element discretization in the virtual work expression (25), the magnetic flux density and the strain can be expressed in terms of vector potential energy and the displacement variables.

(31) $$\begin{array}{l} B_{e}=\nabla \times A_{e}=\nabla \times (N^{A}q_{e}^{A})=C_{e}q_{e}^{A}\\ S_{e}=\nabla u_{e}=\nabla (N^{u}q_{e}^{u})=G_{e}q_{e}^{u} \end{array}$$

The elements in the matrices $C_{e}$ and $G_{e}$ are the derivatives of the local coordinate system ξ about the global coordinate system x, which is assumed to be the discrete form of curl and gradient. For linear elements, Jacobean matrix ${𝒥}_{e}$ is a constant matrix. Then, matrix $C_{e}$ and $G_{e}$ does not depend on ξ. Assuming that magnetic field be divided into $N^{A}$ elements, the mechanical field is discretized into $N^{u}$ elements. The virtual work balance expression can be shown as follows:

(32) $$\begin{array}{l} \sum_{e=1}^{N^{A}}\left(\int_{\Delta }^{}H\cdot C_{e}\delta q_{e}^{A}J_{e}d\Delta +\int_{\Delta }^{}\sigma_{e}N^{A}\frac{\partial q_{e}^{A}}{\partial t}\cdot N^{A}\delta q_{e}^{A}J_{e}d\Delta \right)\\ \;\;\;\;\;\;\;\;\;\;\;\;\;\;\;\;\;\;\;\;\;\;\;\;\;\;\;\;\;\;\;=\sum_{b=1}^{N_{S}^{A}}\int_{\Delta }^{}H_{T}\cdot N^{A}\delta q_{b}^{A}J_{e,S}d\Delta_{S}+\sum_{e=1}^{N^{A}}\int_{\Delta }^{}J_{s,e}N^{A}\delta q_{e}^{A}J_{e}d\Delta \\ \sum_{e=1}^{N^{u}}\left(\int_{\Delta }^{}T\cdot G_{e}\delta q_{e}^{u}J_{e}d\Delta +\int_{\Delta }^{}\rho_{e}N^{u}\frac{\partial^{2}q_{e}^{u}}{\partial t^{2}}\cdot N^{u}\delta q_{e}^{u}J_{e}d\Delta +\int_{\Delta }^{}c_{e}N^{u}\frac{\partial q_{e}^{u}}{\partial t}\cdot N^{u}\delta q_{e}^{u}J_{e}d\Delta \right)\\ \;\;\;\;\;\;\;\;\;\;\;\;\;\;\;\;\;\;\;\;\;\;\;\;\;\;\;\;\;\;\;=\sum_{b=1}^{N_{S}^{u}}\int_{\Delta_{S}}^{}t_{b}\cdot N^{u}\delta q_{b}^{u}J_{e,S}d\Delta_{S} \end{array}$$

Because the influence of gravity and Lorentz force in the magnetostrictive device can be neglected, body force could be ignored [25]. Where subscript b denotes the number of boundary elements, $N_{S}^{A}$ and $N_{S}^{u}$ represents the number of boundary elements of the magnetic field and the mechanical field, respectively. Integral $\int_{\Delta }^{}J_{e}d\Delta$ means element integration in the global coordinates. Then, the element volume can be described as (33).

(33) $$\int_{\Delta }^{}J_{e}d\Delta =\int_{-1}^{1}\int_{-1}^{1}\int_{-1}^{1}\det(\frac{\partial x}{\partial \xi })d\xi_{1}d\xi_{2}d\xi_{3}=V_{e}$$

The integral $\int_{\Delta_{S}}^{}J_{S,b}d\Delta_{S}$ means boundary element integration in the global coordinate, which represents the element area.

(34) $$\int_{\Delta_{S}}^{}J_{S,b}d\Delta_{S}=\int_{-1}^{1}\int_{-1}^{1}\det\left[\begin{array}{cc} \frac{\partial x_{i}}{\partial \xi_{i}} & \frac{\partial x_{i}}{\partial \xi_{j}}\\ \frac{\partial x_{j}}{\partial \xi_{i}} & \frac{\partial x_{j}}{\partial \xi_{j}} \end{array}\right]d\xi_{i}d\xi_{j}=A_{b}$$

### 2.5. Magneto Mechanical Coupling Nonlinear Model

Magneto mechanical coupling relationship [26] of the magnetostrictive actuator system can be described by the piezomagnetic equation:

(35) $$\begin{array}{l} B=\mu^{T}H+dT\\ S=d^{T}H+S^{H}T \end{array}$$

where $\mu^{T}$ is permeability tensor at constant stress; $S^{H}$ is flexibility coefficient tensor at constant magnetic field intensity; and d and S are the piezomagnetic tensor and strain, respectively.

The above equations show that the total magnetic flux density consisted of two parts. The first part shown in the first term denotes the magnetic flux density $\mu^{T}H$ that is induced by the applied magnetic field, and the second terms represents magnetic flux density dT that is caused by the application of mechanical stress. Similarly, the total strain is also formed with two terms, where the first term is the magnetic strain $d^{T}H$ that is caused by applied magnetic field, while the second one denotes the elastic strain $S^{H}T$, which is caused by the applied stress. Inside the giant magnetostrictive materials, its strain is immediately relevant with the magnetic intensity, stress state, and its material properties. The actuator’s output displacement and output force are the result of mutual coupling of the magnetic and elastic fields.

The above eigenvalue equations can be expressed in incremental form as follows:

(36) $$\begin{array}{l} \Delta H=a\Delta B-b\Delta S\\ \Delta T=-b^{T}\Delta B+c\Delta S \end{array}$$

The increments of the magnetic field and the stress field are combined with the solution result calculated by the finite element through the Equation (36). Solving from the known initial solution, the pre-compression t, surface magnetic field strength $H_{T}$, and source current density $J_{S}$ are all in incremental form. Replacing the discrete variables in the Equation (31) with Formula (36) and (37) can be derived as follows:

(37) $$\begin{array}{l} H_{e}=a_{e}C_{e}q_{e}^{A}-b_{e}G_{e}q_{e}^{u}\\ T_{e}=-b_{{}^{e}}^{T}C_{e}q_{e}^{A}+c_{e}G_{e}q_{e}^{u} \end{array}$$

The above equation can be substituted into Equation (32), and the symbols of the integral variables in the equation are defined as follows:

(38) $$\begin{array}{l} k_{e}^{u}=\int_{\Delta }G_{e}^{T}c_{e}G_{e}J_{e}d\Delta \\ k_{e}^{A}=\int_{\Delta }C_{e}^{T}a_{e}C_{e}J_{e}d\Delta \\ k_{e}^{u,A}=\int_{\Delta }C_{e}^{T}b_{e}G_{e}J_{e}d\Delta \\ d_{e}^{A}=\int_{\Delta }{\left(N^{A}\right)}^{T}\sigma_{e}N^{A}J_{e}d\Delta \\ d_{e}^{u}=\int_{\Delta }{\left(N^{u}\right)}^{T}c_{e}N^{u}J_{e}d\Delta \\ m_{e}=\int_{\Delta }{\left(N^{u}\right)}^{T}\rho_{e}N^{u}J_{e}d\Delta \\ f_{b}^{u}=\int_{\Delta_{S}}{\left(N^{u}\right)}^{T}tJ_{e,S}d\Delta_{S}\\ f_{b}^{A}=\int_{\Delta_{S}}{\left(N^{A}\right)}^{T}H_{T}J_{e,S}d\Delta_{S}\\ f_{e}^{J}=-\int_{\Delta }{\left(N^{A}\right)}^{T}J_{s,e}J_{e,S}d\Delta \end{array}$$

According to the definition of the symbols shown above, the virtual work balance expression of the magnetic field and the mechanical field can be written as follows:

(39) $$\begin{array}{l} \sum_{e=1}^{N^{A}}(d_{e}^{A}\frac{\partial q_{e}^{A}}{\partial t}+k_{e}^{A}q_{e}^{A}-k_{e}^{u,A}q_{e}^{u})\cdot \delta q_{e}^{A}=\sum_{e=1}^{N_{A}}f_{e}^{J}\cdot \delta q_{e}^{A}+\sum_{e=1}^{N_{S}^{A}}f_{b}^{A}\cdot \delta q_{b}^{A}\\ \sum_{e=1}^{N^{u}}(m_{e}\frac{\partial^{2}q_{e}^{u}}{\partial t^{2}}+d_{e}^{u}\frac{\partial q_{e}^{u}}{\partial t}+k_{e}^{u}q_{e}^{u}-{\left(k_{e}^{u,A}\right)}^{T}q_{e}^{A})\cdot \delta q_{e}^{u}=\sum_{b=1}^{N_{S}^{u}}f_{b}^{u}\cdot \delta q_{b}^{u} \end{array}$$

Reorganizing the above equation with variational principle, it can be written into a matrix form:

(40) $$\left[\begin{array}{cc} 0 & 0\\ 0 & m_{e} \end{array}\right]\left[\begin{array}{c} {\ddot{q}}_{e}^{A}\\ {\ddot{q}}_{e}^{u} \end{array}\right]+\left[\begin{array}{cc} d_{e}^{A} & 0\\ 0 & d_{e}^{u} \end{array}\right]\left[\begin{array}{c} {\dot{q}}_{e}^{A}\\ {\dot{q}}_{e}^{u} \end{array}\right]+\left[\begin{array}{cc} k_{e}^{A} & -k_{e}^{u,A}\\ -{\left(k_{e}^{u,A}\right)}^{T} & k_{e}^{u} \end{array}\right]\left[\begin{array}{c} q_{e}^{A}\\ q_{e}^{u} \end{array}\right]=\left[\begin{array}{c} f_{e}^{J}+f_{b}^{A}\\ f_{b}^{u} \end{array}\right]$$

As it can be seen from the above equation, the mass matrix is singular and only the mass of mechanical field is included. As the giant magnetostrictive actuator is excited by a frequency lower than MHz under normal working conditions, the electric displacement vector can be neglected. As a result, the second derivative term of the vector potential energy will be zero. The damping matrix is mainly derived from the material’s internal damping $d_{e}^{u}$ and the eddy current damping $d_{e}^{A}$ (the eddy current is depended on the conductivity). In the stiffness matrix, $k_{e}^{A}$ depends mainly on the magnetic permeability and it characterizes the capability of the magnetic excitation system. The coupling matrix $k_{e}^{u,A}$ characterizes the capability of the magnetic field converted into mechanical energy by the mechanical traction vector $f_{b}^{u}$ under the surface traction and capability of the mechanical energy converted into the magnetic energy at the magnetic field load vector $f_{e}^{J}+f_{b}^{A}$.

The incremental form of the coefficient relationship (36) can be solved by the free energy model. Its constitutive model can be written as a function of magnetic flux density B and the mechanical strain S about the magnetic field intensity H and the mechanical stress T, and Equation (35) is derivative. The coefficients can be expressed in the form of the binary function’s derivative:

(41) $$\begin{array}{l} a=\frac{\partial B}{\partial H}(H_{0},T_{0})\\ b=\frac{\partial B}{\partial T}(H_{0},T_{0})\\ b^{T}=\frac{\partial S}{\partial H}(H_{0},T_{0})\\ c=\frac{\partial S}{\partial T}(H_{0},T_{0}) \end{array}$$

Coefficients in above formula can be written into matrix form shown as (42):

(42) $$\left[\begin{array}{cc} a & b\\ b^{T} & c \end{array}\right]=\left[\begin{array}{cc} \frac{\partial B}{\partial H} & \frac{\partial B}{\partial T}\\ \frac{\partial S}{\partial H} & \frac{\partial S}{\partial T} \end{array}\right]$$

For the giant magnetostrictive materials, the differences of the lattice orientation, the driving magnetic field, and the stress will cause the changes of the parameter matrix values. It is assumed that the pre-pressure of the actuator is large enough to dominate the lattice anisotropy so that the changes of the parameter matrix caused by anisotropy ignoring can be ignored. Then, the magnetostrictive material is considered to be isotropous. According to equation of $M=B/\mu_{0}-H$ and the scalar free energy model, the above matrix values can be solved respectively by M.

## 3. Calculation of the Coupling Model of Giant Magnetostrictive Actuator (GMA)

A giant magnetostrictive actuator is a new type of precision actuator, which is based on the magnetostriction deformation effect of magnetostrictive material rod when the input magnetic field changes. Giant magnetostrictive actuators is designed according to the properties of giant magnetostrictive materials. In this paper, we perform finite element simulation analysis on the established magnetomechanical coupling model.

### 3.1. The Structure and Working Principle of the Giant Magnetostrictive Actuator

**Figure 2:**
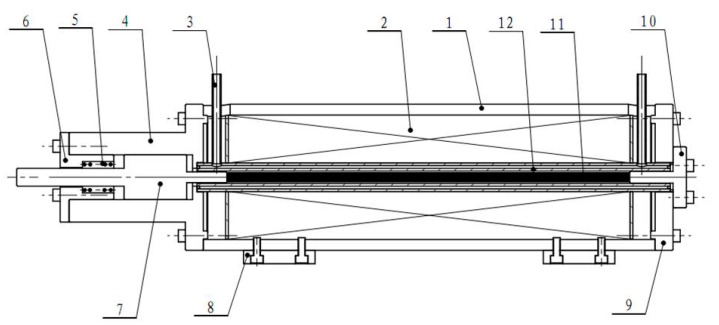
Structural diagram of giant magnetostrictive actuator with non-uniform coils. 1. shell; 2. coil; 3. inlet and outlet; 4. output shaft sleeve; 5. preloading spring; 6. front-end cover; 7. output shaft; 8. fixed plate; 9. backend cover; 10. small end cover; 11. giant magnetostrictive material (GMM) rod; 12. coil bobbin outer tube.

The structure of giant magnetostrictive actuator is shown in Figure 2; it is mainly composed of giant magnetostrictive material rod, coil, coil bobbin outer tube, output shaft, front-end cover, rear end cover, shell, inlet and outlet, preloading spring, and fixing plate. In order to reduce the magnetic flux leakage and rise the magnetic field intensity of the center axis of the giant magnetostrictive material rod, the closed magnetic circuit consists of the giant magnetostrictive material rod, the output shaft, the shell, and the rear end cover. The output shaft, the front end cover, and the preloading spring form a preloading mechanism together, and pre-pressure is given to the giant magnetostrictive material rod in the pre-experiment period so that the giant magnetostrictive material rod obtains a relatively larger axial output strain under the action of the magnetic field. Then, greater output displacement and force are obtained. At the same time, the giant magnetostrictive rod is exerted with a certain pre-pressure to keep it working on a state of compression and prevent material’s fragmentation caused by its high brittleness. The coil bobbin outer tube composes a water-cooled cavity. The water inlet and outlet are used as the lead-in and lead-out holes to allow the overall system of the actuator to carry out water circulation, thereby keeping the temperature of the GMM rod within a certain range to suppress thermal deformation.

The working principle of the giant magnetostrictive actuator is that the driving coil generates a magnetic field under the effect of the direct current signal, which causes the giant magnetostrictive rod to induce magnetostrictive deformation to move the output shaft and realize the output of the force and the displacement. Using the method of adjusting the driving DC signal can change the amplitude of the magnetic field. By this way, we can obtain the output of the displacement and the force in different sizes.

### 3.2. Finite Element Simulation of the Coupling Model of Giant Magnetostrictive Actuator (GMA)

In this study, the MATLAB2009a software demo (which is a commercial mathematics software produced by MathWorks, MA, USA) is used to program the model, and a demo version of COMSOL 4.0a software demo (which eveloped by the COMSOL group who founded in Stockholm, Sweden) [17] is used to simulate the coupling model of giant magnetostrictive actuator. The analysis process includes the following: Firstly, COMSOL finite element simulation software is used to construct the geometric model and mesh the actuator, and the coefficient matrix is iterated through the MATLAB software. Secondly, both two are invoked to complete the coupled analysis of the material model and the dynamic model. In theory, the magnetic field of the coil fills the whole space. In the finite element simulation, a larger closed cuboid air field is built to substitute the space. The giant magnetostrictive actuator is placed in the air field, and the boundary of air field is set to be magnetic insulation.

**Figure 3:**
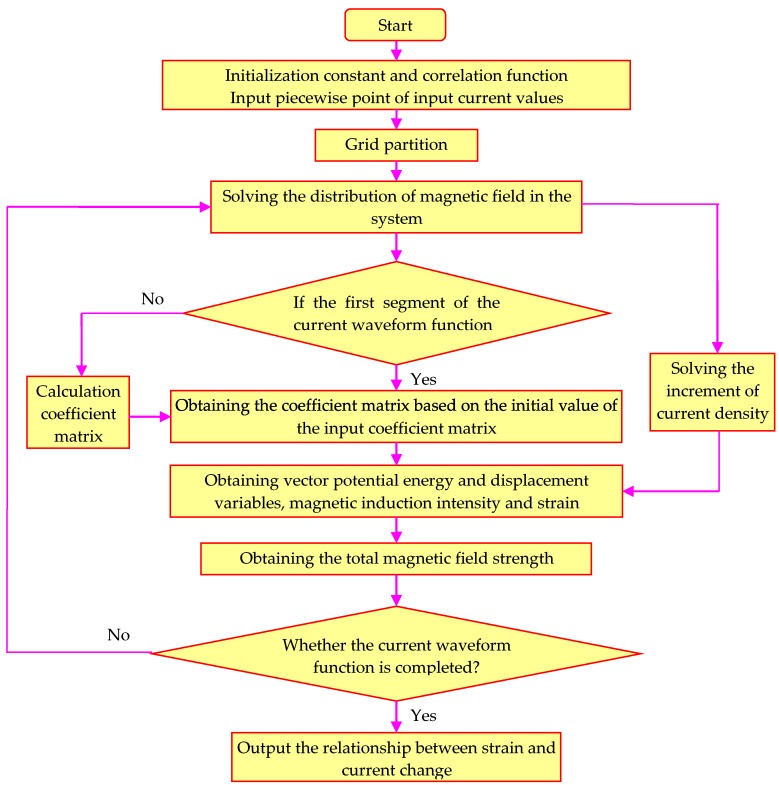
Flowchart of the model solving.

The diameter of the GMM rod is 10 mm, and its length is 450 mm. The material of the shell is chosen to be 45 steel. The diameter of single wire of driving coil is 1 mm, and its height is 22 mm. The total number turns of winding coil is 9570 turns. The solution process of the model is shown in Figure 3: Firstly, the current values are inputted and the model is meshed. Secondly, the increment of the initial magnetic field strength can be obtained by the input of initial coefficient matrix. Thirdly, the coefficient matrix is updated by solving the model, where the increments of magnetic induction and strain should be calculated so that further new incremental magnetic field intensities can be obtained. The cycle is repeated until the current waveform function is terminated.

### 3.3. Simulation Result of Magneto Mechanical Coupling Model of Giant Magnetostrictive Actuator (GMA)

**Figure 4:**
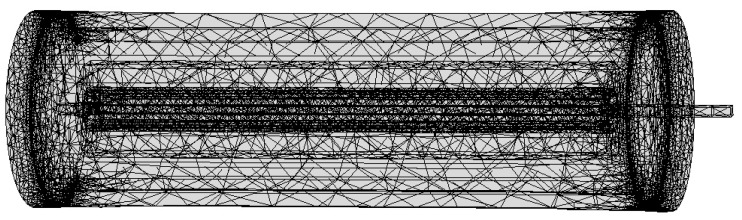
Meshing of a giant magnetostrictive actuator.

A meshing strategy is chosen in the finite analysis as it is shown in the literature [19,21]. In order to reflect the output characteristics of giant magnetostrictive material rods in a better way, the giant magnetostrictive material rods and output shaft are meshed more intensively (Figure 4). Because the driving magnetic circuit and driving coil are of a symmetrical structure, and both of them have large sizes, the mesh is relatively sparse so that the number of elements and the number of degrees of freedom in the model solving process can be reduced. Then, the operation efficiency can be improved.

**Figure 5:**
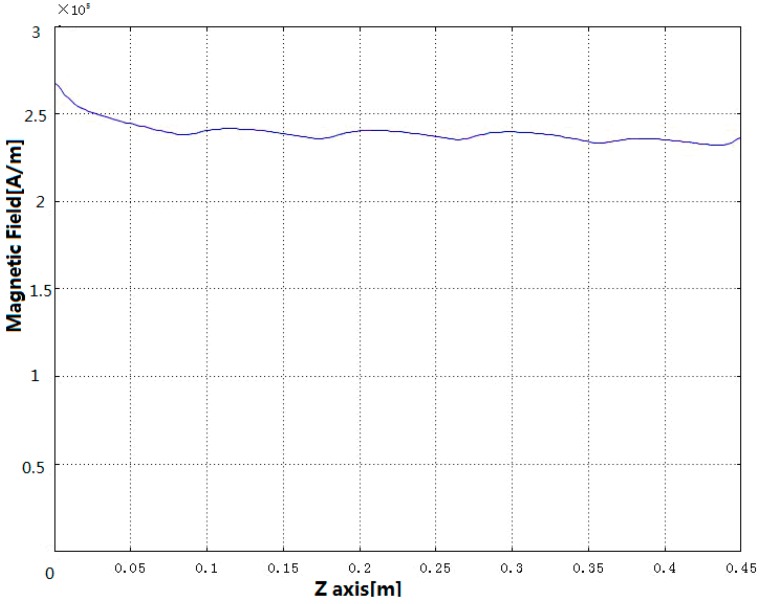
The distribution of magnetic field in the center axis of GMM rod.

The maximum input electric current is set to be 5 A, and preloading is 10 MPa. The axial magnetic field distribution curve is shown in Figure 5, from which we can see that GMM rod axial magnetic field strength is around 250 kA/m.

**Figure 6:**
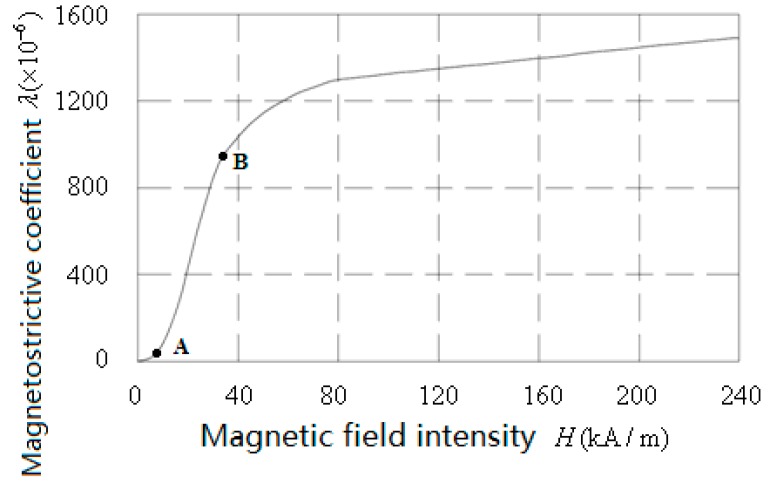
The λ−H curve of the GMM rod.

Figure 6 presents GMM rod’s λ−H curve provided by Gansu Star Company (pre-pressure is 10 MPa), as shown in the figure, the linear working area of GMM is located between the AB segments. According to the design requirements, the intensity of the magnetic field distributed on the GMM rod should be not less than 40 kA/m, taking into consideration of flux leakage and other factors, we know that the giant magnetostrictive actuator designed in this paper can fully meet the design requirements.

**Figure 7:**
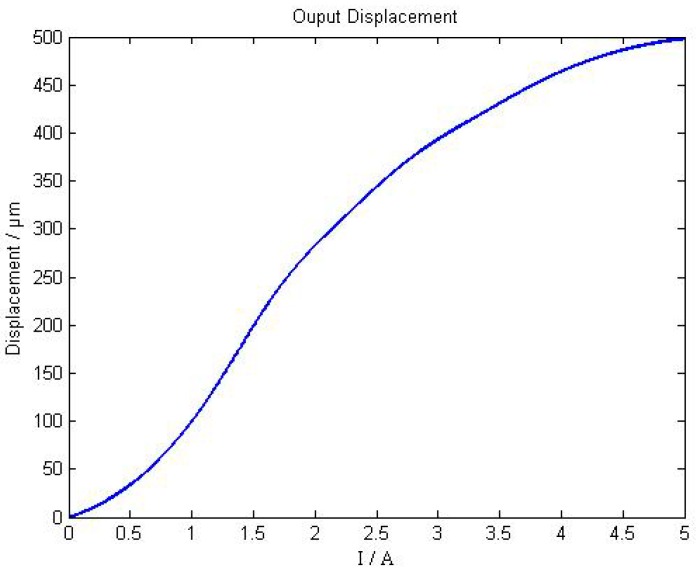
Axial output displacement curve of giant magnetostrictive actuator.

**Figure 8:**
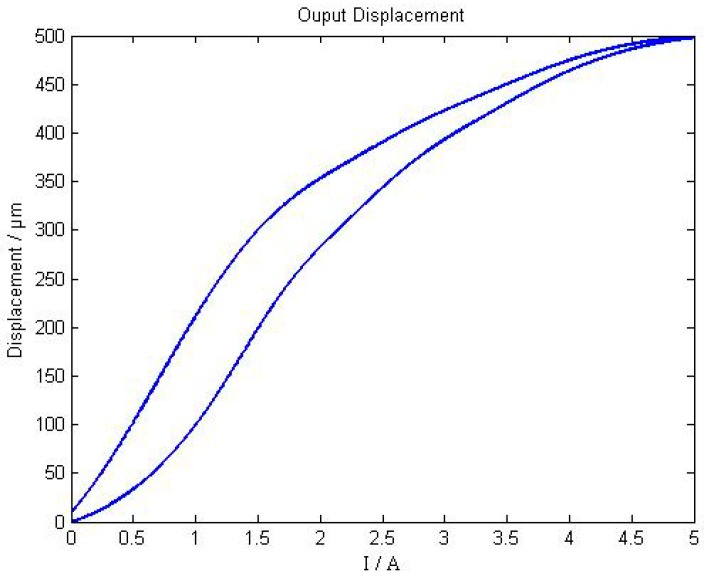
Hysteretic loop of a giant magnetostrictive actuator.

When the input current increases monotonically from 0 A to 5 A, the axial output displacement curve of the giant magnetostrictive actuator is shown as Figure 7. When the input current is from 0 A to 5 A, then from 5 A to 0 A, the hysteretic loop of the giant magnetostrictive actuator is shown in Figure 8.

As shown in Figure 7, the maximum output displacement is 498 μm , and when the input current is changed from 0 A to 3 A, the linearity of giant magnetostrictive actuator is much better. When the input current is greater than 3 A, the output displacement presents worse linearity. From Figure 8, the worst hysteresis happens in situations when the current is around 1 A, and the hysteresis is 25.02%.

## 4. Testing and Experimental Research on Giant Magnetostrictive Actuator

Based on the finite element analysis of the magneto mechanical coupling model of the giant magnetostrictive actuator [27], a system platform for performance testing of the developed giant magnetostrictive actuator is set up, and the experiment research on the output displacement characteristics of giant magnetostrictive actuator is conducted. Through comparison of the experimental and simulation results, the output displacement and hysteresis of the two systems will be comprehensively analyzed.

### 4.1. Experimental Platform

**Figure 9:**
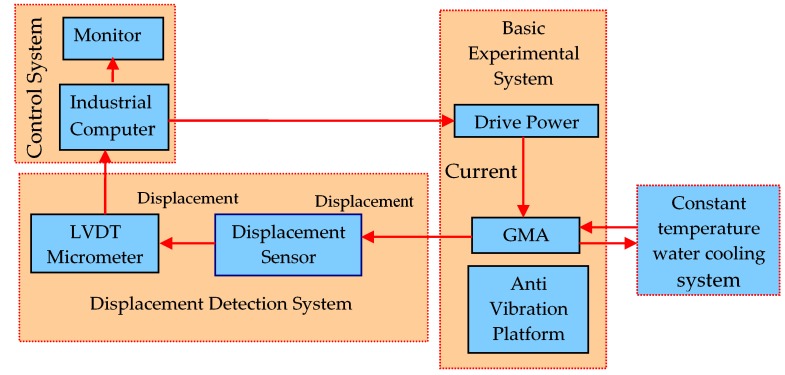
Overall experimental system platform. GMA—giant magnetostrictive actuator.

The overall experimental system platform is shown in Figure 9, it consists of a giant magnetostrictive actuator, vibration isolation platform, constant current source, control system, displacement detection system, and constant temperature water cooling system.

**Figure 10:**
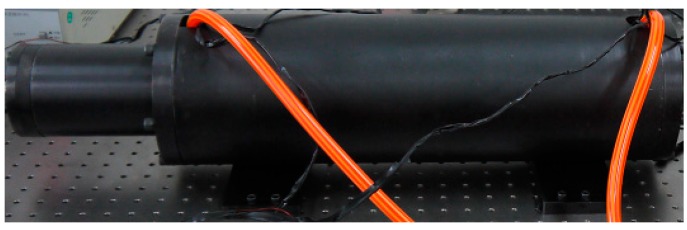
Experimental prototype of a giant magnetostrictive actuator.

In this study, a prototype of a giant magnetostrictive actuator was designed and manufactured as shown in Figure 10. For the system platform, which is built for the prototype, industrial PC (PC is an abbreviation for personal computer) is taken as its control system. Firstly, the input voltage signal is entered from the industrial PC to controllable DC (DC is an abbreviation for direct-current) voltage constant-current power supply named DH1716A (Beijing Dahua Radio Instrument Factory, Beijing, China), the constant current source receives the signal and outputs the corresponding current. The driving coil generates a magnetic field under the effect of the input current. Because of the action of the magnetic field, GMM rod generates deformation and outputs displacement. The displacement signal is detected by the LVDT (LVDT is an abbreviation for linear variable differential transformer, which is a linear displacement sensor) micrometer (manufactured by Bosch Precision Measurement and Control Company, which was founded in Stuttgart, Germany, the version is MDS-L-0500-M6-1A), whose accuracy reached the micrometer-level, and which will finally be sent to industrial PC.

The fluxmeter used in the experiment is the HT701 digital fluxmeter produced by Shanghai Hengtong Magnetism Technology Company, Ltd.,which was founded in Shanghai, China. Its measuring range can be divided into six steps (0.2, 0.5, 1, 2, 4, 8) × 20 wb, and the measurement accuracy can reach ±1% of the full scale. The HAP-100 series air-cushion vibration isolation platform manufactured by Shanghai Tiannuo Electromechanical Company, Ltd. (which was founded in Shanghai, China) was used to meet the vibration isolation requirements in the experimental process.

The application test software developed based on visual basic (VB) language runs directly on the industrial PC. It communicates with the constant current source and the LVDT micrometer through the data serial port line. By controlling the output current magnitude of the constant current source, the real-time displacement value displayed from the micrometer can be read, and the test data will be output as TXT (TXT is an abbreviation for text document format) text document for archiving.

The constant temperature hydrocooling system consists of four parts: cooling water tank, temperature sensor, cooling fan, and heating rod. The temperature controller used in the experiment is an ATC-800 Microprocessor Temperature Controller made in Shanghai Jingchuang electric appliance manufacturing Company. Ltd., Shanghai, China, whose measuring accuracy is ±1 °C and whose indicating range is 0 °C–45 °C.

### 4.2. Experimental Research on Giant Magnetostrictive Actuator

In this study, a magnetostrictive experimental study of a giant magnetostrictive actuator is performed based on an experimental system of a giant magnetostrictive actuator. Through the acquired measurement data, and with the help of the analysis part of the test software we have already written, the useful information is extracted from the measured data and it will be analyzed.

(1)   The experiment of output characteristic

**Figure 11:**
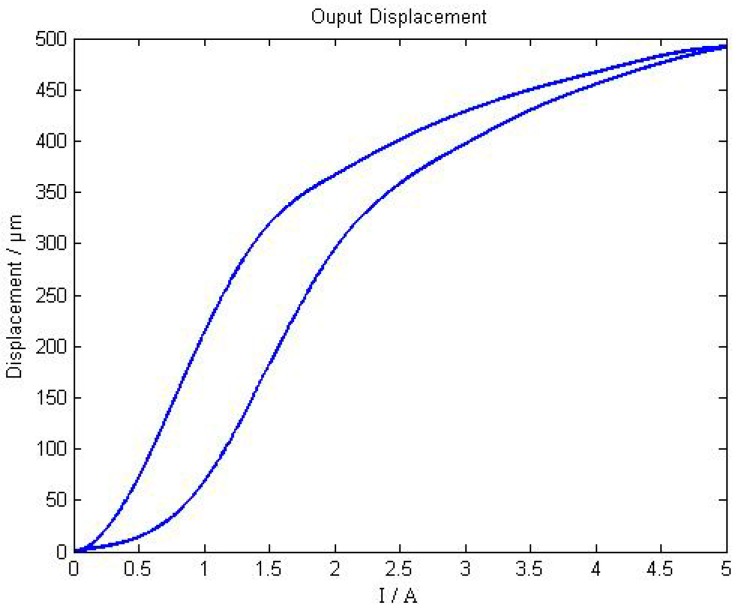
Hysteresis loop under the action of current 5 A and preloading 10 MPa.

When the pre-pressure is 10 MPa and the input current is from 0 A to 5 A, then from 5 A to 0 A, the hysteretic loop of the giant magnetostrictive actuator is shown in Figure 11. When the input current increases from 0 A to 5 A monotonically, the relationship between output displacement and current is in the lift range. When the input current decreases from 5 A to 0 A monotonously, the relationship between the output displacement and the current is in the return stage, and the output displacement exhibits a significant hysteretic effect throughout the entire return stage.

The hysteresis is defined as follows:

(43) $$e=\frac{\max\left|y_{2i}-y_{1i}\right|}{\max(y_{1})}\times 100\%$$

where $y_{1}$ is the displacement during the lift range, $y_{1i}$ denotes the displacement during the lift stage (when the current is i), and $y_{2i}$ represents the displacement during the return stage (when the current is i).

From the equation above, the hysteresis is 29.74%.

(2)   Comparison between experimental results and simulation results

**Figure 12:**
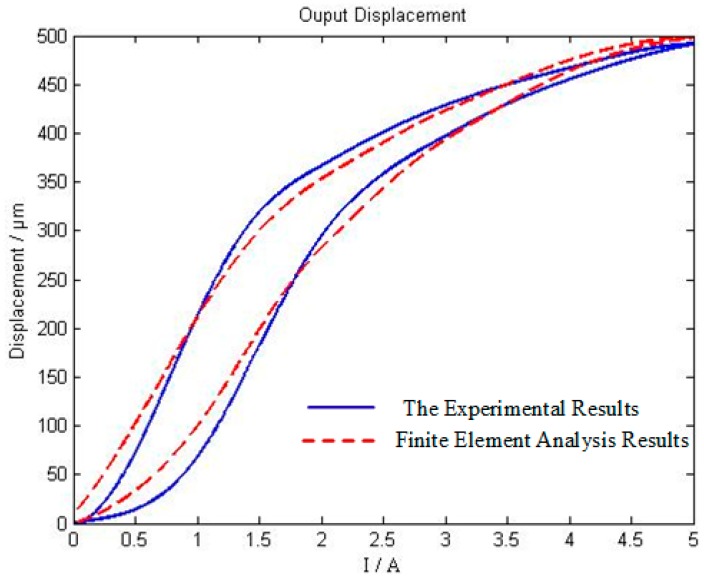
Contrast diagram of hysteretic loop between experiment and finite element analysis.

The experimental results are compared with the simulation results (as shown in Table 1). The results of finite element analysis and the results of output data are basically consistent, and most of the relative errors are stable at less than 15%, which means the established finite element model of magneto mechanical coupling is reasonable and the analysis result is also accurate. This model presents certain reference effects in the design process of giant magnetostrictive actuators. Figure 12 shows the comparison chart of the hysteretic loop between the experimental results and the finite element analysis results (the real line shows the experimental results, and the dotted line shows the finite element analysis results).

It can be seen from Figure 12 that the experimental results (the blue curve) are basically coincident with the hysteretic loops of the finite element analysis (the red dotted curve). When the current value is lower than 2.0 A, the deviation between the experimental curve and the simulation curve is large; when the current value exceeds 4 A, the deviation is also significantly larger, the current interval with the smallest deviation is between 2.0 A and 4.0 A. The reason for this phenomenon is that there is an optimal working current range for the giant magnetostrictive material, under the action of outside the optimal working current range, the hysteresis effect is obvious, and the displacement output error is large. In the actual application process, it should try to make it work in the best current range (the optimal working current range needs to be drawn by experiment). At the same time, there is a coincidence error between the finite element simulation curve and the experimental curve, which is because of the measurement error of the LVDT micrometer and the influence of the temperature change for the giant magnetostrictive material during the experiment. The giant magnetostrictive material has an optimum working temperature range, although the constant temperature treatment is adopted, the magnetic properties of the giant magnetostrictive material change when the temperature value changes because of the influence of heat generated by the current. At the beginning, the giant magnetostrictive material works at a constant temperature of 25 °C, when the current is entered for a period of time, the internal temperature of the rod of the giant magnetostrictive material increases, the hysteresis characteristic changes, and the linearization of the displacement output is improved. When the working time is slightly longer, the temperature rise is larger, the increase of the temperature affects the hysteresis effect, and curvature of the experimental curve of the displacement output is inconsistent with the simulation curve. The optimal working temperature and the optimum working current range require further post-experimental studies. At the same time, the multi-physics coupling characteristics of mechanical giant magnetostrictive materials consisting of the hysteresis characteristics, the mechanical properties, temperature, and others need to be further studied based on the research of this paper.

Comparing the experimental hysteresis with the finite element analysis hysteresis, the experimental result is 29.74%, the finite element analysis result is 25.02%, and the relative error is 15.87%. This shows that the proposed three-dimensional magneto mechanical coupling finite element model can describe the dynamic performance of the giant magnetostrictive actuator accurately and can play an auxiliary role in structural optimization.

## 5. Conclusions

For the giant magnetostrictive actuator, in this paper, at first, the eigen model of giant magnetostrictive material is constructed in combination with the free energy hysteresis model, and according to the fundamental equations of magnetic field and mechanical field of the giant magnetostrictive actuator, the three-dimensional nonlinear magnetic machine coupling model under weak solution is derived from MAXWELL equation and Newton’s second law. Then, the axial magnetic field distribution of the giant magnetostrictive actuator is simulated using COMSOL finite element simulation software and MATLAB software, and the magnetic induction intensity value and output strain value with the current change controlled by the magnetic field are obtained, and the simulation results are verified experimentally. The results show that the experimental results of the output displacement and hysteresis of the giant magnetostrictive actuator are basically consistent with the simulation results, and the relative error is 15.87%, these show that the magnetic field strength on the central axis of the giant magnetostrictive actuator falls within the linear working area of the GMM under the action of the solenoid magnetic field (working current linear range is 2.0 A–4.0 A), and the magnetic circuit design is reasonable, the three-dimensional magnetic machine coupling model of the giant magnetostrictive actuator based on free energy hysteresis characteristics is designed to meet the input and output characteristics of the giant magnetostrictive actuator. The research conclusions have important guiding significance for the structural optimization of giant magnetostrictive actuator and the practical application of giant magnetostrictive materials.

## Figures and Tables

**Table 1:** Comparison between the finite element model analysis and experimental results.

Electric Current (A)	Output Displacement (μm)		Error (%)
	Finite Element Simulation Results	Experimental Results	
0	0	0	0
0.5	27.94	14.61	47.71
1	92.65	67.52	27.12
1.5	205.88	182.65	11.28
2	282.35	293.99	3.96
2.5	348.53	358.52	2.79
3	398.53	397.4	0.28
3.5	432.35	430.27	0.48
4	466.18	455.42	2.31
4.5	493.12	475.99	3.47
5	498.09	491.63	1.30
